# Risk Stratification of Indeterminate Thyroid Nodules (TIR3A vs. TIR3B): Impact of NIFTP Reclassification in a Surgical Cohort

**DOI:** 10.3390/cancers18091481

**Published:** 2026-05-05

**Authors:** Pietro De Luca, Giulia Chiappino, Luca de Campora, Anna Sambito, Angelo Camaioni, Claudio Viti

**Affiliations:** 1 Laboratory of Neuromotor Physiology, IRCSS Foundation Santa Lucia, 00179, Rome, Italy; 2 Otolaryngology Department, San Giovanni and San Filippo Hospital, 67051 Avezzano, Italy; 3 Otolaryngology Department, Santa Rosa Hospital, 01100 Viterbo, Italy; 4 Head and Neck Department, Nostra Signora della Mercede Clinic, 00198 Rome, Italy

**Keywords:** indeterminate thyroid nodules, fine-needle aspiration cytology, TIR3A/TIR3B classification, NIFTP, thyroid cancer

## Abstract

Background: Indeterminate thyroid nodules classified as TIR3 according to the SIAPEC-IAP classification represent a heterogeneous group with variable malignancy risk, complicating clinical management. The reclassification of non-invasive follicular thyroid neoplasm with papillary-like nuclear features (NIFTP) has further influenced risk estimates. Methods: We conducted a retrospective single-center study including adult patients who underwent ultrasound-guided fine-needle aspiration cytology followed by thyroid surgery between January 2016 and September 2025. Cytological diagnoses were classified according to the SIAPEC-IAP system and correlated with final histology. Ultrasound risk stratification was performed according to EU-TIRADS criteria. Risk of malignancy (ROM) and cytology–histology concordance were assessed, including the impact of NIFTP reclassification. Results: A total of 228 patients were included, with an overall malignancy rate of 27.6%. TIR3A nodules showed a significantly lower ROM than TIR3B (15.6% vs. 48.7%), decreasing to 12.5% and 41.4%, respectively, after exclusion of NIFTP. Cytology–histology agreement was substantial (κ = 0.72). Ultrasound features associated with malignancy included microcalcifications, irregular margins, taller-than-wide shape, and EU-TIRADS ≥4. The BRAF V600E mutation was significantly associated with malignant histology. Conclusions: TIR3A and TIR3B nodules exhibit markedly different malignant potential and should not be managed as a homogeneous group. NIFTP significantly modifies malignancy risk estimates and represents an important source of potential overtreatment. Integration of cytological subclassification with ultrasound and molecular findings improves preoperative risk stratification and supports personalized management of indeterminate thyroid nodules.

## Simple Summary

Thyroid nodules are very common in the general population, but only a small proportion are malignant. Fine-needle aspiration cytology is widely used to evaluate these nodules and guide clinical management. However, some nodules show indeterminate cytological features, making it difficult to decide whether surgery is necessary. In the Italian classification system, these lesions are subdivided into two categories with different levels of risk. In this study, we analyzed patients who underwent thyroid surgery to evaluate how well these cytological categories predict the final diagnosis. We also assessed the impact of a recently reclassified tumor type that behaves in a very indolent manner but can still lead to surgical treatment. Our results show that combining cytological classification with ultrasound findings and molecular markers may improve risk assessment and help clinicians choose more appropriate management strategies, potentially reducing unnecessary surgery.

## 1. Introduction

Thyroid nodular disease is one of the most common endocrine disorders worldwide, with a prevalence reaching 50–60% in the adult population when assessed by high-resolution cervical ultrasound [1]. Despite this high prevalence, most thyroid nodules are benign, and only a minority—estimated between 5% and 15%—harbor malignant disease [2]. The main clinical challenge is therefore to identify nodules requiring surgical treatment while avoiding unnecessary procedures in patients with benign lesions.

Fine-needle aspiration cytology (FNAC) represents the cornerstone of preoperative evaluation of thyroid nodules. This minimally invasive and cost-effective technique allows cytomorphological assessment of follicular cells and provides reliable risk stratification in most cases [3]. The introduction of standardized reporting systems has substantially improved diagnostic reproducibility and communication between cytopathologists and clinicians.

The Bethesda System for Reporting Thyroid Cytopathology (TBSRTC) established six diagnostic categories associated with defined risks of malignancy (ROM) and management recommendations [4]. In Italy, the SIAPEC-IAP classification, updated in 2014, adapted the Bethesda framework and introduced a clinically relevant subdivision of indeterminate lesions into two subclasses: TIR3A (low-risk indeterminate lesion) and TIR3B (high-risk indeterminate lesion) [5]. In general terms, TIR3A roughly corresponds to Bethesda category III (AUS/FLUS), whereas TIR3B is conceptually similar to Bethesda category IV (follicular neoplasm/suspicious for follicular neoplasm).

Despite the widespread adoption of the SIAPEC-IAP system, the reported ROM for TIR3A and TIR3B nodules remains highly variable across institutions, reflecting differences in patient selection, surgical indications, and histopathological interpretation. Moreover, the reclassification of non-invasive follicular thyroid neoplasm with papillary-like nuclear features (NIFTP) has further modified malignancy risk estimates, particularly within indeterminate cytological categories.

In this context, real-world data integrating cytological, histological, ultrasound, and molecular findings are needed to refine risk stratification and optimize surgical decision-making. The present study therefore aims to evaluate the predictive value of TIR3A and TIR3B cytological diagnoses in a large single-center surgical cohort, assess cytology–histology concordance, and determine the impact of NIFTP reclassification on malignancy risk estimates.

## 2. Materials and Methods

### 2.1. Study Design

This retrospective observational study was conducted in the Department of Otorhinolaryngology at Santa Rosa Hospital, a tertiary referral center in Viterbo, Italy. The study was designed to assess cytology–histology correlation in thyroid nodules classified as TIR3A or TIR3B according to the SIAPEC-IAP 2014 classification [2].

All consecutive adult patients undergoing ultrasound-guided FNAC followed by thyroid surgery were included (between January 2016 and September 2025).

### 2.2. Study Population and Diagnostic Assessment

Inclusion criteria were as follows: (1) age ≥ 18 years; (2) FNAC diagnosis of TIR3A or TIR3B; (3) availability of definitive histological diagnosis. Exclusion criteria were as follows: (1) Persistently non-diagnostic cytology (TIR1); (2) absence of surgical treatment; (3) incomplete clinical or histological data.

All patients underwent high-resolution cervical ultrasound (US) performed by experienced operators using linear high-frequency probes (10–15 MHz), and FNAC was performed under ultrasound guidance using 23–25 gauge needles (at least two passes were obtained). Surgical specimens were fixed in 10% buffered formalin and reviewed according to World Health Organization (WHO) Classification of Endocrine Tumors (5th edition, 2022) [6]. According to current consensus diagnostic criteria, NIFTP cases were considered non-malignant.

Ultrasound assessments were performed independently by two experienced radiologists blinded to cytological and histopathological findings, thereby minimizing interpretation bias. Disagreements were resolved through consensus review, and persistent discrepancies were adjudicated by a third senior radiologist according to EU-TIRADS criteria [7]. When required for diagnostic clarification, immunohistochemical analyses were performed on formalin-fixed paraffin-embedded tissue sections using established markers (thyroglobulin, TTF-1, calcitonin, CEA, HBME-1, galectin-3, and CK19). Molecular analyses were conducted separately using polymerase chain reaction (PCR)-based techniques for the detection of specific gene mutations.

Cytological and histological specimens were evaluated by dedicated endocrine pathologists using standardized diagnostic criteria. Histological assessment was performed independently of the cytological diagnosis whenever possible, and cases with diagnostic uncertainty were reviewed jointly to achieve consensus.

### 2.3. Statistical Analysis

Statistical analysis was performed using IBM SPSS v26 and R Foundation v4.0. Continuous variables were described as the mean ± standard deviation or the median (interquartile range (IQR)), depending on the distribution, as verified using Shapiro–Wilk tests. Student’s *t*-tests or Mann–Whitney *U* tests were used to compare groups (TIR3A vs. TIR3B), while categorical variables were compared using chi-squared or Fisher's exact tests. For the analysis of diagnostic performance among indeterminate nodules, TIR3B cytology was considered a positive test result for malignancy, whereas TIR3A was considered a negative test result. Final histological diagnosis served as the reference standard.

Diagnostic performance measures including sensitivity, specificity, positive predictive value (PPV), negative predictive value (NPV), and overall accuracy were calculated with 95% confidence intervals.

A multivariable logistic regression analysis was performed to identify independent preoperative predictors of malignancy among indeterminate nodules. Only TIR3A and TIR3B cases were included. The dependent variable was malignant histology at final diagnosis (yes/no). Independent variables included cytological subclassification (TIR3B vs. TIR3A), age at surgery (continuous), sex (male vs. female), EU-TIRADS category (≥4 vs. <4), and presence of suspicious ultrasound features (microcalcifications, irregular margins, or taller-than-wide shape). Variables were selected based on clinical relevance and availability prior to surgery. Results are presented as odds ratios (ORs) with 95% confidence intervals (CIs).

Receiver operating characteristic (ROC) curve analysis was performed to evaluate the ability of cytological classification to discriminate between benign and malignant histology. For this analysis, malignant histology included papillary thyroid carcinoma, follicular carcinoma, and other malignant tumors, whereas benign lesions and NIFTP were considered non-malignant according to current consensus criteria. The analysis included nodules across the cytological spectrum (TIR2–TIR5). The optimal cut-off point was determined using the Youden index, and corresponding sensitivity and specificity were calculated.

### 2.4. Ethical Considerations

This study was conducted in accordance with the Strengthening the Reporting of Observational Studies in Epidemiology (STROBE) guidelines for observational studies [8]. According to national regulations for retrospective observational studies, formal ethics committee approval was waived. The study adhered to the principles outlined in the Helsinki Declaration. All data were entered into an electronic database.

## 3. Results

### 3.1. Study Population

Between January 2016 and September 2025, a total of 228 patients met the inclusion criteria, comprising 174 females (76.7%) and 54 males (23.3%). All patients underwent thyroid fine-needle aspiration cytology (FNAC) followed by surgical treatment with definitive histopathological examination. The mean age at surgery was 62.9 years for women and 65.8 years for men (*p* > 0.05).

Overall, thyroid carcinoma was diagnosed in 63 patients (27.6%). The frequency of carcinoma was comparable between sexes but slightly higher in males (29.6%) than in females (27.0%). Due to the predominance of female patients, women accounted for 66.2% of all carcinoma cases, whereas men represented 33.8%.

Among patients younger than 55 years, women were more numerous (44 vs. 11 men), with a mean age of 43.7 years. However, the incidence of carcinoma in this age group was higher in males (45.5%) than in females (25.0%), suggesting a greater relative risk among younger men despite their lower representation in the cohort.

Among patients aged ≥55 years, sex-related differences in carcinoma frequency were less pronounced, with a higher absolute number of female cases but a more balanced malignancy rate between sexes.

### 3.2. Distribution of Cytological Categories and Risk of Malignancy

The distribution of cytological diagnoses according to the SIAPEC-IAP classification showed a progressive increase in risk of malignancy (ROM) from TIR2 to TIR5 (Table 1).

TIR2 nodules showed a malignancy rate of 9.5%, whereas TIR3A and TIR3B nodules demonstrated rates of 6.7% and 22.4%, respectively. TIR4 and TIR5 categories were associated with a high risk of malignancy, with carcinoma confirmed in 50% and 90.9% of cases, respectively.

### 3.3. Impact of NIFTP Reclassification

A relevant proportion of cases corresponded to non-invasive follicular thyroid neoplasm with papillary-like nuclear features (NIFTP), which significantly influenced malignancy estimates.

Histological examination identified 10 NIFTP cases initially classified as TIR3A, 19 cases initially classified as TIR3B, and 1 case initially classified as TIR5.

NIFTP lesions, characterized by papillary-type nuclear features without capsular or vascular invasion, are currently considered non-malignant neoplasms with indolent behavior. Their reclassification as non-malignant resulted in a reduction of the corrected ROM for each cytological category.

Two additional TIR5 cases corresponded to hyalinizing trabecular adenomas, rare benign tumors known to mimic papillary thyroid carcinoma both cytologically and histologically.

Non-diagnostic categories (TIR1 and TIR1c), not included in the main analysis due to their small number, accounted for 0.6% and 2.5% of cases, respectively. The single TIR1 case proved to be a follicular variant of papillary thyroid carcinoma with vascular invasion and distant vertebral metastases at presentation, underscoring that a non-diagnostic cytology result cannot be considered reassuring.

### 3.4. Final Histological Diagnosis

The distribution of histological diagnoses is summarized in Table 2. Benign lesions predominated overall. Among malignant tumors, papillary thyroid carcinoma was the most common subtype, followed by follicular carcinoma and follicular variants of papillary carcinoma. NIFTP represented a substantial proportion of cases (12.7%).

Considering NIFTP as non-malignant, the overall corrected ROM was 27.6%.

For indeterminate nodules:

- TIR3A ROM: 15.6% (10/64);

- TIR3B ROM: 48.7% (40/82).

After exclusion of NIFTP from malignant lesions:

- Corrected TIR3A ROM: 12.5%;

- Corrected TIR3B ROM: 41.4%.

These values fall within the ranges reported by current SIAPEC-IAP recommendations.

### 3.5. Cytology–Histology Concordance

Cohen’s kappa coefficient was calculated after collapsing cytological categories into benign and malignant groups to assess clinically meaningful agreement with histological diagnosis while minimizing category imbalance.

The overall concordance was substantial, with κ = 0.72 (95% CI: 0.60–0.84), corresponding to substantial agreement according to the Landis and Koch classification [9]. Agreement was highest for extreme categories (TIR2 and TIR5), whereas concordance was lower for intermediate categories, particularly TIR3A and TIR3B. When restricting the analysis to indeterminate categories (TIR3A vs. TIR3B), agreement with final histology remained moderate (κ = 0.46; 95% CI 0.31–0.61), reflecting the intrinsic biological overlap between low- and high-risk indeterminate lesions.

### 3.6. Diagnostic Performance of TIR3A and TIR3B

Among indeterminate cytology categories, histological outcomes differed markedly between TIR3A and TIR3B nodules.

TIR3A nodules were predominantly benign on final histology, yielding a high negative predictive value (NPV 84.4%), consistent with a low-risk category. In contrast, TIR3B nodules showed nearly equal distributions of benign and malignant outcomes, corresponding to a positive predictive value (PPV) of 48.7% and indicating an intermediate-to-high risk of malignancy.

Overall diagnostic performance of indeterminate cytology (TIR3A and TIR3B combined) for predicting malignancy was as follows:

- Sensitivity: 80.0% (95% CI 69.3–89.1)

- Specificity: 65.1% (95% CI 56.7–72.9)

- PPV: 48.7% (95% CI 40.3–57.0)

- NPV: 84.4% (95% CI 77.1–90.1)

- Accuracy: 67.5%

These findings suggest the markedly different risk profiles of the two subcategories, with TIR3A behaving as a low-risk group and TIR3B as a clinically relevant indeterminate category requiring careful management.

### 3.7. ROC Curve Analysis and Multivariable Logistic Regression

ROC curve analysis confirmed the overall discriminatory ability of the SIAPEC-IAP cytological classification for predicting malignancy (AUC 0.78, 95% CI 0.69–0.86), indicating good diagnostic performance across the cytological spectrum. For this analysis, malignant histology included papillary thyroid carcinoma, follicular carcinoma, and other malignant tumors, whereas benign lesions and NIFTP were considered non-malignant according to current consensus criteria. The optimal cut-off value identified by the Youden index corresponded to the threshold between low-risk and high-risk cytological categories, yielding a sensitivity of 80.0% and a specificity of 65.1% for the prediction of malignant histology.

In the multivariable logistic regression analysis, no independent preoperative predictors of malignancy were identified. Cytological subclassification (TIR3B vs. TIR3A) showed higher odds of malignancy but did not reach statistical significance (OR 1.17, 95% CI 0.59–2.33; *p* = 0.650). Age (OR 1.006 per year, 95% CI 0.983–1.030; *p* = 0.616) and male sex (OR 0.95, 95% CI 0.47–1.94; *p* = 0.889) were also not significantly associated with malignant histology.

### 3.8. Ultrasound and Molecular Features

Ultrasound risk stratification was performed according to the EU-TIRADS classification proposed by the European Thyroid Association. Ultrasound features significantly associated with malignancy included microcalcifications (*p* = 0.004), irregular margins (*p* = 0.012), taller-than-wide shape (*p* = 0.021), and EU-TIRADS ≥ 4 (*p* < 0.001).

Molecular testing was performed in a subset of patients in whom additional diagnostic clarification was considered clinically indicated. Among the tested cases (n = 152), BRAF V600E mutations were detected in 22 cases, RAS mutations in 11 cases, and TERT promoter mutations in 2 cases. Because molecular testing was not systematically performed in all patients, these results should be interpreted with caution due to potential ascertainment bias.

## 4. Discussion

Indeterminate thyroid nodules continue to pose a significant diagnostic and management dilemma despite advances in cytology and imaging. In particular, the TIR3 category of the Italian reporting system encompasses a heterogeneous group of lesions with widely variable risks of malignancy, making clinical decision-making difficult [10,11]. The present study provides a comprehensive analysis of cytological, histological, ultrasound, and molecular features in a surgically treated cohort, highlighting factors associated with malignancy and potential tools for risk stratification. However, in the multivariable logistic regression analysis, cytological subclassification did not emerge as an independent predictor of malignancy. This finding should be interpreted cautiously, as the model was likely underpowered due to the limited sample size and the relatively small number of malignant events within the indeterminate subgroup.

In our series, the overall malignancy rate among surgically treated nodules was high, reflecting the inherent selection bias toward patients undergoing thyroidectomy. This finding is consistent with previous surgical cohorts, where malignancy rates are substantially higher than those reported in unselected FNAC populations [5]. Therefore, our results should be interpreted in the context of a preselected population with clinical or radiological indications for surgery.

Our results are broadly consistent with current recommendations from both the American Thyroid Association and the European Thyroid Association [3,7]. These guidelines advocate a personalized approach to indeterminate nodules, integrating cytological findings with ultrasound risk stratification and clinical factors. In particular, both societies recognize that nodules with low-risk cytology and benign ultrasound features may be managed conservatively, whereas nodules with higher-risk profiles should be considered for surgical intervention. The higher malignancy rate observed in TIR3B nodules in our study supports recommendations favoring diagnostic lobectomy in this subgroup, especially when additional risk factors are present.

A key finding of this study is the markedly different risk of malignancy between TIR3A and TIR3B nodules. TIR3A lesions showed a relatively low malignancy rate, whereas TIR3B nodules demonstrated a substantially higher risk, confirming the clinical utility of subclassification within the indeterminate category. These results align with previous studies validating the Italian consensus system, which reported malignancy risks of approximately 5–15% for TIR3A and 15–40% for TIR3B [12,13,14]. The higher malignancy rate observed in our TIR3B group further supports current recommendations favoring surgical management for these lesions, particularly in the presence of suspicious ultrasound features.

The impact of NIFTP deserves particular attention. In our cohort, a considerable proportion of indeterminate lesions corresponded to NIFTP, and their reclassification as non-malignant significantly reduced apparent malignancy rates. NIFTP therefore functions as a biological confounder in the estimation of malignancy risk for indeterminate cytology, as it requires surgical excision for definitive diagnosis despite its indolent behavior. This issue is especially relevant in indeterminate categories, where NIFTP represents a substantial fraction of surgically treated lesions and may contribute to overtreatment if not adequately considered in preoperative risk assessment [15,16].

Ultrasound findings played a significant role in malignancy prediction [17,18]. Features such as microcalcifications, irregular margins, and a taller-than-wide shape were significantly associated with carcinoma, in accordance with established evidence [19]. Moreover, higher EU-TIRADS categories were significantly correlated with malignant histology. The EU-TIRADS system developed by the European Thyroid Association has demonstrated good diagnostic performance in multiple studies, with reported sensitivities exceeding 80% for high-risk categories [7]. Our findings support its usefulness in refining risk assessment in indeterminate nodules and support its integration into clinical algorithms.

Molecular testing provided additional diagnostic information. The detection of the BRAF V600E mutation was significantly associated with classical papillary thyroid carcinoma and with higher cytological risk categories, particularly TIR3B. This is consistent with the well-established role of BRAF as a driver mutation in papillary thyroid carcinoma and a marker of malignancy in indeterminate nodules [20,21,22,23]. RAS mutations were less specific, reflecting their association with follicular-patterned lesions, including benign adenomas, NIFTP, and follicular carcinoma [12]. TERT promoter mutations were rare but occurred exclusively in malignant tumors, in line with their association with aggressive disease [24,25].

Cytology–histology concordance analysis demonstrated substantial agreement when categories were collapsed into clinically relevant benign versus malignant groups. This approach reduces the impact of category imbalance and reflects real-world clinical decision-making. Agreement was highest for extreme categories (TIR2 and TIR5), while intermediate categories showed lower concordance, underscoring the intrinsic uncertainty of indeterminate cytology. Similar patterns have been reported across different classification systems, including Bethesda [4]. The SIAPEC-IAP classification system used in the present study is the standard of care in Italy and shares broad conceptual alignment with The Bethesda System for Reporting Thyroid Cytopathology (TBSRTC), which is more widely adopted in Anglo-American centres. The two systems are broadly comparable: TIR2 broadly corresponds to Bethesda II (benign), TIR3A to Bethesda III (atypia of undetermined significance/follicular lesion of undetermined significance, AUS/FLUS), TIR3B to Bethesda IV (follicular neoplasm/suspicious for follicular neoplasm, FN/SFN), TIR4 to Bethesda V (suspicious for malignancy), and TIR5 to Bethesda VI (malignant). The ROM values observed in the present cohort for TIR3A (15.6%) are consistent with those reported for Bethesda III in surgical series, while the ROM for TIR3B (48.7%) falls in the upper range reported for Bethesda IV, likely reflecting the inherent enrichment for suspicious lesions in surgically treated cohorts [4,26,27]. A direct head-to-head comparison between systems was not feasible in the present study, as all institutional FNAC reports were issued according to SIAPEC-IAP criteria; however, the conceptual concordance between the two frameworks supports the broader applicability of our findings to international clinical practice.

Receiver operating characteristic analysis confirmed the overall diagnostic accuracy of cytology, with high specificity but moderate sensitivity. This reflects the conservative nature of indeterminate diagnoses, which aim to minimize false positives at the expense of increased surgical referrals. In clinical practice, cytology should therefore be interpreted in conjunction with ultrasound findings, clinical risk factors, and molecular results.

The inclusion of nodules across the entire cytological spectrum allowed evaluation of diagnostic performance beyond the TIR3 category alone. However, the most clinically relevant insights derive from the indeterminate subgroup, where management remains controversial. Our data suggest that combining cytological subclassification, ultrasound risk stratification, and molecular testing can substantially improve preoperative risk assessment.

The management of indeterminate thyroid nodules increasingly relies on a multimodal approach integrating cytology, ultrasound assessment, molecular testing, and clinical factors. Our findings support a risk-adapted strategy in which cytological subclassification defines baseline risk, ultrasound features refine anatomical suspicion, and molecular markers provide biological insight. Selected TIR3A nodules with low-risk imaging features may be considered for conservative management within a shared decision-making framework, whereas TIR3B nodules—particularly those with high EU-TIRADS scores or positive molecular alterations—are more appropriately managed with diagnostic surgery. Such an integrated strategy may reduce unnecessary operations while ensuring timely treatment of clinically significant malignancies.

The increasing complexity of thyroid nodule management has prompted growing interest in artificial intelligence-assisted diagnostic approaches. Recent comprehensive reviews have examined the application of machine learning, deep learning, and transformer-based models to thyroid carcinoma detection and classification, demonstrating promising performance across imaging and cytological modalities [27,28]. While such approaches hold significant potential for augmenting conventional diagnostic workflows, their integration into routine clinical practice remains limited by challenges related to dataset heterogeneity, model interpretability, and the absence of prospective validation in real-world settings. The present study, grounded in established cytological, histological, and molecular criteria within a well-defined institutional cohort, contributes to this landscape by providing the type of granular, multimodal clinical data that future integrative diagnostic frameworks will require for contextual validation.

### Limits of the Study

Several limitations must be acknowledged. First, the retrospective design introduces potential biases, including incomplete data and lack of standardized management protocols. Second, as only surgically treated nodules were analyzed, the total number of TIR3A and TIR3B nodules diagnosed during the study period but managed conservatively was not systematically recorded in the institutional database. Consequently, the magnitude of the potential selection bias cannot be precisely quantified. Third, molecular testing was not uniformly performed in all cases, limiting the generalizability of those findings. Finally, the single-center nature of the study may restrict external validity. 

Despite these limitations, the study has notable strengths, including a relatively large sample size, comprehensive multimodal evaluation, and detailed histological classification incorporating NIFTP. The integration of cytological, radiological, and molecular data reflects contemporary multidisciplinary management of thyroid nodules.

From a clinical perspective, our findings support a risk-adapted approach to indeterminate thyroid nodules. TIR3A lesions without suspicious ultrasound features may be suitable for conservative management and surveillance, whereas TIR3B nodules—especially those with high EU-TIRADS scores or positive molecular markers—should be considered for surgery. Future prospective studies are needed to refine predictive models and to evaluate the role of emerging molecular panels and artificial intelligence–based ultrasound analysis.

## 5. Conclusions

Indeterminate thyroid nodules classified as TIR3A and TIR3B exhibit substantially different malignant potential and should not be managed as a homogeneous group. TIR3A lesions generally behave as low-risk nodules and may be suitable for active surveillance, whereas TIR3B nodules carry a clinically relevant risk of malignancy that often justifies surgical intervention, especially when high-risk ultrasound features or molecular alterations are present.

NIFTP represents a significant contributor to apparent malignancy rates in indeterminate cytology and may lead to overtreatment if not appropriately considered.

A multimodal evaluation integrating cytology, ultrasound, and molecular data appears essential for optimal risk stratification and individualized management of patients with indeterminate thyroid nodules.

## Figures and Tables

**Table 1:** Distribution of surgically treated thyroid nodules according to SIAPEC-IAP cytological categories and corresponding final histological diagnosis. Data include the total number of cases per category, malignant and benign outcomes, and the calculated risk of malignancy (ROM).

Cytological Category	Total Cases	Malignant	Benign	ROM (%)
TIR2	36	3	33	9.5
TIR3A	64	10	54	6.7
TIR3B	82	40	42	22.4
TIR4	24	12	12	50.0
TIR5	22	20	2	90.9

Note: One additional case with non-diagnostic cytology (TIR1) was identified during the study period and proved to be a follicular variant of papillary thyroid carcinoma with vascular invasion on final histology; this case was excluded from Table 1 as it did not contribute to the cytological categories under analysis.

**Table 2:** Distribution of histological diagnoses among surgical patients.

Histological Diagnosis	Cases	Percentage %
Papillary thyroid carcinoma (including variants and microcarcinomas)	41	18
Follicular carcinoma/follicular variant of papillary carcinoma	9	3.9
Medullary thyroid carcinoma	5	2.3
Anaplastic thyroid carcinoma (ATC)	1	2.2
Oncocytic thyroid carcinoma	2	
Poorly differentiated thyroid carcinoma (PDTC)	2	
High grade differentiated carcinoma (HGDTC)	3	1.3
Follicular adenoma/adenomatoid lesion	7	3.1
Nodular hyperplasia/multinodular goiter/colloid goiter	42	18.4
Chronic thyroiditis (including Hashimoto thyroiditis)	39	17.1
Adenomatoid nodules not classified elsewhere	48	21
NIFTP	29	12.7

## Data Availability

Data are available from the corresponding author upon reasonable request.
